# How do different cell populations orchestrate myelin regeneration?

**DOI:** 10.1042/BST20231085

**Published:** 2025-06-23

**Authors:** Sara Grassi, Alessandro Prinetti

**Affiliations:** Department of Medical Biotechnology and Translational Medicine, Via Fratelli Cervi 93, 20090 Segrate (Milano),University of Milan, Milan, Italy

**Keywords:** multiple sclerosis, remyelination, rHIgM22, sphingolipids, sphingosine 1-phosphate

## Abstract

Approximately 35 in 100,000 people are affected by diseases associated with loss of myelin, generally described as demyelinating diseases. Demyelinating diseases encompass many different pathological conditions characterized by heterogeneous and sometimes disease-specific etiopathological mechanisms. While several approaches aimed at ameliorating the symptoms and the progression of some of these diseases exist, the most effective cure for all demyelinating diseases would be regeneration of lost myelin. Myelin regeneration occurs spontaneously in the central nervous system in response to myelin damage but is inefficient for a variety of reasons, especially in human patients. In this review, we will discuss the contributions of different cell populations to the creation of conditions permissive for effective remyelination and to the formation of new myelin after injury. Moreover, we would like to highlight the importance of sphingolipids in the network of interactions between these cell populations. Mutations in genes encoding sphingolipid metabolic enzymes (such as *GALC*) represent a major risk factor for multiple sclerosis, and alterations in sphingolipid metabolism in specific cell types contribute to myelin damage. On the other hand, sphingolipid signaling, in particular through sphingosine 1 phosphate, directly affects the process of myelin regeneration, with distinct effects on different cellular populations.

## Introduction

Myelin facilitates nerve impulse conduction in myelinated axons, allowing higher conduction speed and lower energy consumption [1,2]. Thus, loss of myelin, as occurs in both genetically determined and acquired demyelinating diseases, initially causes loss of saltatory conduction, but in the medium to long term deeply affects axonal integrity and ultimately axonal survival, as clearly demonstrated by the marked axonal loss observed in multiple sclerosis (MS) during the chronic evolution of the disease [3]. More recently, myelin has also been shown to play a relevant role in the regulation of neuronal circuits [4]. Unsurprisingly, demyelination results in devastating and, in the long run, irreversible dysfunction of the nervous system. The events leading to demyelination are heterogeneous (even if inflammation is a common trait in most acquired demyelinating diseases), making it difficult to envision a common therapeutic strategy aimed at preventing or stopping myelin loss [5-8]. On the other hand, while some disease-modifying therapies aimed at containing myelin damage or minimizing its consequences are available, regeneration of damaged or lost myelin would be the most effective approach to cure virtually all demyelinating diseases. In this sense, regenerative medicine has recently been explored as a possible tool to induce myelin regeneration [9-12]. Cell therapy undoubtedly holds promise for the treatment of demyelinating diseases through myelin regeneration. However, the mammalian brain possesses an intrinsic ability to regenerate damaged myelin. Studies on animal models clearly indicate that triggering myelin regeneration is the brain’s standard response to myelin loss and that the process is effective enough to allow full and quick recovery from experimentally induced damage, thus largely preventing neurodegeneration. Unfortunately, human demyelinating diseases are characterized by a much higher level of complexity than is replicated in animal models with experimentally induced myelin lesions. Even if there is convincing evidence that myelin regeneration is also triggered in human patients with lesions, the outcome of the regenerative process is in general less favorable than in animal models and varies greatly from patient to patient.

Understanding the reasons for this failure of myelin regeneration would pave the road for the development of treatments able to boost the ability of the brain to regenerate myelin. One of the factors that limits the effectiveness of spontaneous myelin regeneration is the presence of non-permissive conditions at the lesion sites. One of these is the presence of damaged myelin debris that negatively affects oligodendrocyte precursor cell (OPC) maturation. Myelin debris can be removed by microglial phagocytic activity, which can be effectively boosted by treatment with the remyelination-inducing rHIgM22 antibody [13]. Another nonpermissive factor is the strong and sustained inflammation at lesion sites that is characteristic of acquired demyelinating diseases, most notably MS. In addition, the formation of glial scars, consequent to astrocyte activation in response to inflammation, strongly inhibits the formation of new myelin. However, astrocytes activated to a neuroprotective phenotype also reduce the detrimental effects of inflammation and can positively affect myelin regeneration. Thus, the effectiveness of myelin regeneration depends on the activation of different cell populations in a way that preserves their beneficial effects while limiting inhibitory signals.

Once the inhibitory signals have been removed or reduced, there is still the question of which cells are responsible for the formation of new myelin, and what are the clues driving them toward myelin-producing phenotypes? OPC-derived oligodendrocytes (and, in some cases, OPC-derived Schwann cells) are usually regarded as the major cells responsible for the formation of new myelin; however, not all OPC subpopulations contribute equally to the generation of myelin-forming oligodendrocytes. Thus, effective myelin regeneration also requires specific signals aimed at recruiting cell populations specifically dedicated to the generation of new myelin after damage or loss.

### Demyelination

Demyelination is the pathological loss of myelin sheaths from around axons. The loss of the myelin coating has two important consequences for the affected axon:

1. The organization of voltage-dependent K^+^ and Na^+^ channels at the iuxtaparanode and node regions is lost, resulting in loss of saltatory conduction. Continuous conduction along the demyelinated axon is made possible by adaptative responses [14] that enable the propagation of the action potential, although at a lower speed. In the long run, however, these responses have negative effects on axonal integrity, due to a high intra-axonal Na^+^ concentration resulting from redistribution of NaV 1.6 channels causing higher than normal currents [15].
2. Myelin is essential for axon survival [16]. Demyelinated axons are prone to degeneration, and marked axonal loss can be observed in MS, likely being a major contributor to the chronic progressive evolution of the disease. Several different aspects contribute to the neuroprotective effect of myelin. Myelin provides trophic metabolic support by shuttling lactate from astrocytes to axons [17,18]. Some myelin proteins play a crucial role in maintaining axonal stability, and myelin-forming oligodendrocytes convey multiple trophic factor signals to the axons [19]. In addition, in demyelinating diseases associated with an inflammatory response, myelin exerts a protective effect against inflammation, likely neutralizing the adverse effects elicited by inflammatory mediators such as those released by microglia.

Demyelinating diseases hallmarked by primary demyelination are often divided into two groups: the leukodystrophies and inflammatory demyelinating diseases.

#### Leukodystrophies

Genetically determined (usually inherited) demyelinating diseases are generically defined as leukodystrophies [20]. Among the leukodystrophies, those characterized by dysfunctions in proteins essential for oligodendrocyte-mediated myelin genesis (e.g., Pelizaeus–Merzbacher disease [21]) can be more properly described as hypomyelinating diseases, since they are characterized by lack of primary myelin formation, rather than by loss of existing myelin [22].

**Figure 1:**
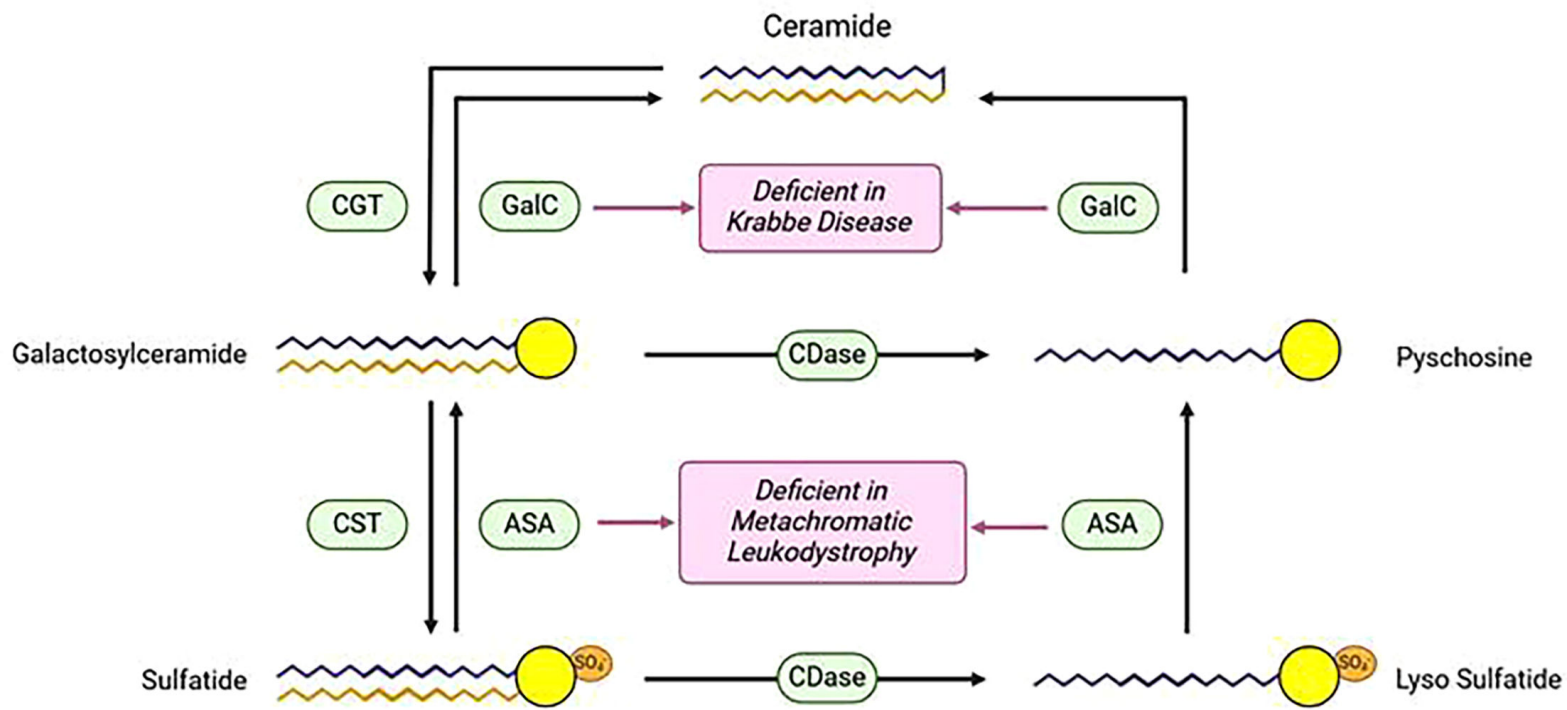
Enzyme defects underlying metachromatic leukodystrophy and Krabbe disease. In healthy OPCs/oligodendrocytes, ceramide in the Golgi is converted into galactosylceramide by ceramide galactosytransferase, and galactosylceramide in turn is the precursor for the synthesis of sulfatide by galactosylceramide sulfotransferase. In the catabolic pathway, sulfatide is cleaved to galactosylceramide by arylsulfatase, and galactosylceramide is subsequently hydrolyzed to ceramide by galactosylceramidase. Metachromatic leukodystrophy is due to deficiency of arylsulfatase A, resulting in the accumulation of undegraded sulfatide and of its deacylated derivative, lysosulfatide, formed by the action of lysosomal ceramidase, whereas Krabbe disease is caused by deficiency in galactosylceramidase with the consequent accumulation of galactosylceramide and of its deacylated metabolite galactosylsphingosine (psychosine). ASA, arylsulfatase; CDase, ceramidase; CGT, ceramide galactosyltransferase; CST, galactosylceramide sulfotransferase; GalC, galactosylceramidase.

Interestingly, some of the leukodystrophies with a demyelinating phenotype are characterized by defects in lipid metabolism, leading to the accumulation of species which are toxic to oligodendrocytes [23]. Metachromatic leukodystrophy [24] and Krabbe disease [25] are caused by genetic defects in lysosomal enzymes; thus, they are also described as lysosomal storage disorders (Figure 1). Metachromatic leukodystrophy is due to lack of arylsulfatase A, resulting in accumulation of the sphingolipid sulfatide, while Krabbe disease is caused by deficiency in galactosylceramidase, with consequent accumulation of galactosylceramide and galactosylsphingosine (psychosine) (Figure 1). In addition, the link between leukodystrophies and altered sphingolipid metabolism has been confirmed by the finding that mutations in the DESG1 gene, encoding the enzyme dihydroceramide desaturase that is responsible for the final step of *de novo* synthesis of ceramide, cause hypomyelinating leukodystrophy [26,27].

#### Inflammatory demyelinating diseases

In this heterogeneous group of diseases, the trigger for myelin damage is an inflammatory insult, and the extent of the demyelination lesions, as well as the extent of the subsequent axonal damage, is highly dependent on the severity of the inflammatory event. Among inflammatory demyelinating diseases, MS has by far the highest prevalence [28,29]. One of the hallmarks of MS is the appearance of focal areas of demyelination, leading to diffused neurodegeneration in the spinal cord and the brain [30,31]. MS is a multifaceted disease, and its etiology is still at least in part undefined. Autoimmune inflammation triggered by autoreactive leukocytes is regarded by most as the cause of myelin damage underlying the onset and clinical phenotype of MS. On the other hand, different environmental [32,33] and genetic risk factors have been associated with the onset of the disease [34,35]. Quite remarkably, several risk factors for MS are not only risk factors for other inflammatory demyelinating diseases but also for neurological disorders that are usually not primarily associated with inflammation/demyelination, including Parkinson’s and Alzheimer’s disease [36]. Of the environmental factors, the ones with the highest association with MS are obesity, tobacco smoking, deficiency of micronutrients (in particular vitamin D), and viral infections [37]. All of these seem to favor a worsening of the inflammatory background of these diseases. Moving to genetic risk factors, genome-wide association studies (nicely summarized in [34]) identified more than 200 variants that associate with increased risk of developing MS. However, none of them are exclusive to MS or necessary or sufficient to cause the disease, highlighting the additive nature of the genetic risk for the development of MS. The functions of several of the mutated alleles corroborated the importance of the immune system in the pathogenesis of MS, but, as the identified genes were not restricted to ones with immune-related functions, the studies also clearly demonstrated the heterogeneity of the pathogenetic mechanisms underlying this disease [38,39]. Indeed, a recent genome-wide association study showed that the age-related MS severity score associates with variations in genes expressed within the central nervous system (CNS), with a cell specificity for the oligodendrocyte lineage, emphasizing the role of protection and repair mechanisms in the progression of MS [40]. Interestingly, *GALC* was among the group of genes identified as risk factors for MS. *GALC* encodes the glycolipid hydrolase galactocerebrosidase, which when deficient above 90% (as occurs in homozygous subjects with two mutated alleles) leads to the development of Krabbe disease (as described above) (Figure 1). Subjects with a single *GALC* mutant allele do not accumulate undegraded substrates and do not develop Krabbe disease. *GALC* ^+/-^ mice showed a phenotype overlapping that of wild-type animals at three months of age, and, when exposed to cuprizone, the extent of oligodendrocyte loss and myelin damage was not significantly different from that observed in wildtype animals. On the other hand, while wildtype animals showed effective remyelination after cuprizone removal, this was impaired in *GALC* ^+/-^ mice at least in part due to impaired clearance of myelin debris by microglia [41,42]. This finding points out the importance of remyelination failure as a risk factor for the onset of MS. Quite remarkably, *GALC* variants have also recently been associated with increased risk for synucleinopathies, including Parkinson’s disease and late-onset schizophrenia [43-45]. This reinforces the notion that variants of genes involved in sphingolipid metabolism represent a common causative background for very heterogeneous neurological disorders.

The number and overall prevalence of classically defined demyelinating diseases would *per se* fully justify interest in mechanisms involved in restoration of missing myelin. However, demyelination occurs even in diseases for which the primary pathological feature is neuronal damage and degeneration, most notably Alzheimer’s disease (AD). In fact, several studies have indicated that axonal demyelination in white matter occurs at very early stages of the disease, even before the onset of clinical symptoms [46]. Demyelination in AD does not seem to be caused by inflammatory or immune-mediated insults but rather by oligodendrocyte impairment due to at least two factors: 1) impaired oligodendrocyte lipid homeostasis, caused by perturbed apoE4-dependent delivery of astrocyte-derived lipids to oligodendrocytes [47,48]; 2) impaired energy metabolism and glycolytic efficiency in differentiating and mature oligodendrocytes [49,50]. These observations are somewhat corroborative of an alternative hypothesis regarding the genesis of MS lesions. Some studies have suggested that MS is indeed a form of oligodendrogliopathy, with oligodendrocyte death leading to microglial activation and further immune responses, both of which would be primarily aimed at clearing myelin debris.

**Figure 2:**
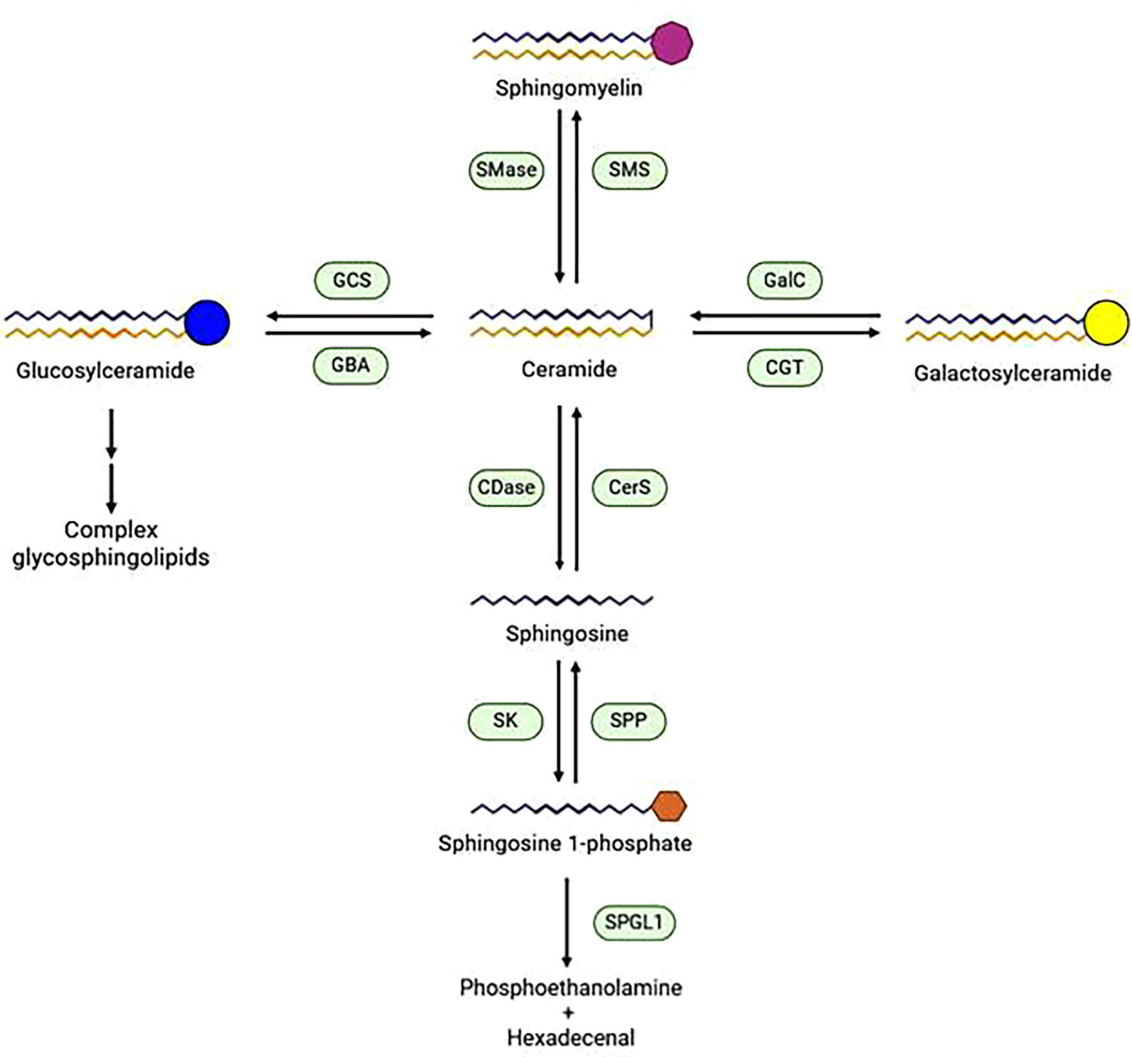
Metabolic pathways involved in the synthesis and degradation of the ‘sphingolipid rheostat’ mediators sphingosine 1 phosphate and ceramide. The levels of sphingosine 1 phosphate and of ceramide in a given cell or tissue are the result of the activities of both biosynthetic and catabolic enzymes, and of a bidirectional flow of molecules between different subcellular locations. The generation of bioactive ceramide is mainly due to the hydrolysis of sphingomyelin by several sphingomyelinases, with different features and diverse subcellular locations, but catabolism of glycosphingolipids can also contribute to the pool of bioactive ceramide. In addition, dihydrosphingosine in the *de novo* biosynthetic pathway (not shown), as well as catabolic sphingosine, can be acylated by different ceramide synthases. Sphingosine 1 phosphate is mainly generated by the action of different sphingosine kinases in different cellular subcompartments. A major source of sphingosine for the synthesis of sphingosine 1 phosphate is ceramide cleavage by ceramidases. Hence, high levels of ceramide usually correspond to low levels of sphingosine 1 phosphate and vice versa (‘the sphingolipid rheostat’). In turn sphingosine 1 phosphate can be degraded by sphingosine 1 phosphate phosphatase or irreversibly cleaved by sphingosine 1-phosphate lyase. CDase, ceramidase; CerS, ceramide synthase; CGT, Ceramide galactosyltransferase; GalC, Galactosylceramidase; GBA, glucocerebrosidase; GCS, glucosylceramide synthase; SK, Sphingosine kinase; SMase, sphingomyelinase; SMS, sphingomyelin synthase; SPP, Sphingosine 1-phosphate phosphatase; SPGL1, Sphingosine 1-phosphate lyase.

Quite remarkably, changes in the levels of the sphingolipid mediators ceramide and sphingosine 1 phosphate (S1P) seem to have great importance in the progression of both AD and MS (Figure 2). Interestingly, these changes affect the properties of multiple cell types involved in the onset of the disease. *In vitro*, ceramide was able to induce apoptosis in cultured oligodendrocytes [51]. *In vivo*, ceramide accumulation (with concomitant decrease of S1P levels) in reactive astrocytes in active lesions of MS in humans and in cuprizone-intoxicated mice affected oligodendrocyte survival and demyelination [52]. The activity of acid sphingomyelinase (ASM) is regarded as the major source for pro-apoptotic ceramide in the context of neurodegenerative and demyelinating diseases. Indeed, genetic deficiency or pharmacological inhibition of ASM results in protective effects [53-57].

In addition, it has been suggested that *de novo* ceramide biosynthesis could significantly contribute to both astrocytopathy and oligodendrocytopathy in MS [52,58]. In fact, it has recently been shown that ceramide synthase 2 expression is significantly increased in primary cultured astrocytes upon treatment with lysophosphatidylcholine, and that this effect is markedly reduced in the presence of anti-demyelinating drugs [59]. Moreover, neuronal-specific deletion of ceramide synthase 5 and 6 attenuated the course of experimental autoimmune encephalomyelitis (EAE) in mice [60]. These findings are broadening the spectrum of sphingolipid metabolic enzymes that could be therapeutic targets in the treatment of demyelinating diseases.

Sphingosine kinase 1 is up-regulated in reactive astrocytes in MS lesions and in cultured rat astrocytes treated with the pro-inflammatory molecule LPS. The contribution of S1P to neuroinflammation in MS is clearly demonstrated by the efficacy of fingolimod in preventing the migration of lymphocytes from the periphery to the CNS following S1P gradients [61,62]. On the other hand, S1P signaling within the CNS contributes to myelin damage, independently of the recruitment of reactive T cells. In EAE, fingolimod efficacy is lost in animals with intact immunological capacity but astrocyte-selective deletion of S1P_1_ [61,63]. Moreover, S1P_2_ activation was shown to be responsible for oligodendrocyte loss and increased demyelination in EAE mice, in part due to increased leakage of the blood–brain barrier [64].

In summary, demyelination is a condition underlying a great number of heterogeneous and diverse neurological diseases, with heterogeneous and diverse mechanisms responsible for the onset and progression of myelin loss. Thus, while on the one hand, approaches aimed at preventing or containing demyelination possess a huge therapeutical potential, it seems unlikely that a common strategy would be effective for a wide range of diseases or even for different forms of the same disease.

### Myelin regeneration

So far, there is no cure available for demyelinating diseases, i.e., therapies aimed at preventing or stopping myelin loss; only disease-modifying therapies aimed at containing myelin damage are currently available [65-67]. The most effective strategy in the case of a demyelinating disease with an inflammatory background envisages the use of drugs able to hamper the immune system response underlying the neuroinflammatory damage [65,68]. In this sense, a successful and intriguing example is represented by the use of fingolimod. Fingolimod (FTY720, commercialized under the proprietary name Gilenya) is one of the few approved oral disease-modifying therapies for the relapsing-remitting form of multiple sclerosis (RR-MS) [69,70]. Fingolimod is a structural analog of sphingosine, and *in vivo* it is phosphorylated by sphingosine kinase(s) to fingolimod-P, a structural analog of sphingosine 1 phosphate (Figure 2). S1P, a soluble signaling sphingolipid, exerts multiple biological functions and is a major regulator of the immune system. In MS, the neuroinflammatory response is mainly sustained by the recruitment and migration of lymphocytes from the periphery to the CNS. Lymphocytes express S1P receptors, and they migrate along S1P gradients. Fingolimod-P is a non-selective S1P receptor agonist, and it acts as a functional antagonist, causing the internalization and degradation of bound S1P receptor [71]. Consequently, fingolimod is effective in MS by preventing the invasion of auto-aggressive T-cells to the CNS. Fingolimod was able to reduce acute inflammatory relapses by 50% in RR-MS [69,72], with strong beneficial quality-of-life effects [67]. However, it had little to no effect on the progression of the disease, despite recent studies showing long-term neuroprotective effects [73]. A fingolimod analog with a similar mechanism of action, siponimod, was approved in 2019 for the treatment of active secondary progressive disease [72], confirming the efficacy of this therapeutic strategy.

Nevertheless, the most effective cure for diseases caused by myelin damage and loss would be the regeneration of lost myelin. Indeed, remyelination has been demonstrated in most animal models of myelin damage, and, most importantly, in human patients. Several pieces of evidence strongly suggest that spontaneous myelin regeneration represents the standard CNS response to myelin damage. For MS, studies of postmortem tissues and, more recently, dynamic imaging in MS patients suggest that myelin regeneration in lesion areas might be more widespread and extensive than previously thought [74-76]. However, remyelination extent and outcome are very heterogenous depending on different factors. Remyelination is strongly limited by the hostile environment of the lesion site, due to the intense inflammatory response [77-79] and the subsequent activation of astrocytes with detrimental effects on myelin regeneration [80]. Focusing on MS, the biggest and foremost difference is the efficiency of the process in animal models of demyelination/MS versus MS patients. While myelin regeneration is very effective in animal models, leading to full recovery in a few weeks after experimental damage, human MS patients show strong variation not only between patients but also between different lesioned areas within the same patient [75,76]. Clearly, none of the available animal models fully recapitulate the complexity of the human disease. This complexity likely results from there being multiple cellular populations involved in the regeneration process with distinct and overlapping triggers and functions, as discussed below.

In humans, the variability of the success of remyelination depends on age, sex, disease duration, stage (inactive vs. active lesions), and location of the lesions (with cortical lesions usually more effectively remyelinated than those in white matter) [74,76,81-83]. The outcome of ineffective myelin regeneration is neurodegeneration that leads to the progression of the disease [77], and thus, strategies aimed at improving spontaneous myelin regeneration as a therapeutic perspective are of paramount importance. Of note, while the pathways leading to myelin loss differ strongly in the different diseases and in different stages of a given disease, as described above, the basic mechanism of myelin regeneration seems to be common, making targeting enhancement of myelin regeneration therapeutically even more appealing as it would be effective for multiple diseases. In this sense, several pieces of evidence suggest that targeting S1P metabolism and signaling at the CNS level has a direct impact on the effectiveness of myelin regeneration. As discussed more in detail below, fingolimod effectiveness in MS is at least in part independent of the recruitment of peripheral immune cells [84]. On the other hand, new-generation S1P receptor modulators with higher selectivity than fingolimod [85] directly affect myelin regeneration by targeting OPCs or oligodendrocytes. Siponimod, selective for S1P_1_ and S1P_5_, exerts direct protective effects on degenerating oligodendrocytes in a S1P_5_-dependent manner with neither lymphocyte modulation nor pro-inflammatory responses from microglia and astrocytes [86]. The pro-regenerative effects of siponimod are characterized by increased density of OLIG2^+^ oligodendrocytes in parallel with reduced density of KiP^+^ proliferating OPCs [87]. Ponesimod, a monoselective modulator of S1P_1_, an S1P receptor expressed at high levels in OPCs, is in turn able to induce myelin regeneration by enhancing OPC differentiation [88]. While S1P receptors represent the most immediate target for pharmacological intervention, clarifying the role of specific enzymes involved in the regulation of the levels of S1P and its metabolites in myelin regeneration is also very important. In this direction, recent findings indicated the importance of sphingosine kinase 2 in allowing effective myelin regeneration [89].

### Is myelin regeneration just OPC business?

Developmental myelination in mammals is largely due to the activity of OPCs. In humans, proliferating OPCs switch to the differentiation program leading to the formation of mature, myelin-forming oligodendrocytes starting from the midgestational period. However, myelination is mainly a postnatal event which is largely completed in early childhood. A consistent population of precursor cells (5-8% of CNS cells, the major population of mitotic cells in the adult brain), commonly referred to as adult OPCs, is present in the adult CNS. Adult OPCs represent a remnant fraction of the developmental OPCs persisting in the adult brain and are apparently able to generate new oligodendrocytes during the entire lifespan. Moreover, they seem to play relevant roles in the physiology of the CNS, e.g., they are responsible for adaptive myelination in response to neuronal activity and are required for learning complex tasks such as those related to behavioral experience [90]. On the other hand, quiescent adult OPCs can be activated upon injury and enter into the myelin regeneration program. Activation of OPCs initially requires active migration to the lesion site and the local induction of a rapid and sustained proliferative response. This recruitment phase is then followed by their differentiation into myelin-forming oligodendrocytes. Recruitment and differentiation of adult OPCs involve both changes in morphology and in gene expression which, at least in part, recapitulate the changes occurring in neonatal OPCs [91,92].

Activated adult OPCs are usually regarded as the major source of new oligodendrocytes in the myelin regeneration process [93]. This is supported by convincing lines of evidence at least in animal models. However, it should be noted that OPCs represent a very heterogeneous cell population [94], e.g., white and grey matter OPCs differ in their proliferation, migration, and differentiation potentials [94,95], and in their electrophysiological properties [96]. Most importantly for the scope of this review, the contribution of the different OPC subpopulations to myelin regeneration after injury is different, and it has been suggested that specific subsets of OPCs might represent the reservoir of cells aimed at myelin regeneration. From this point of view, GPR17, a G protein-coupled receptor transiently expressed in oligodendroglial cells, is emerging as a key gatekeeper for OPCs in myelin regeneration, and GPR17-positive OPCs, widely spread throughout the brain, seem to represent the most relevant pool involved in this event. GPR17 starts to be expressed in early OPCs and reaches a peak in immature O4-positive oligodendrocytes, then it is down-regulated to allow terminal maturation and subsequent myelination [97,98]. Aberrant GPR17 expression has been described both in models of MS and in human MS lesions, leading to blockade of OPCs at intermediate stages and to delay in myelination [99,100]. To become myelinating cells, OPCs and immature oligodendrocytes undergo a deep reorganization of their membrane lipid composition, and GPR17 may be one of the active players in this shift: GPR17 silencing in OPCs leads to up- or down-regulation of approximately 800 genes including key genes related to lipid synthesis and lipid-mediated signaling. In particular, the gene encoding the S1P_3_ receptor is down-regulated, suggesting that GPR17 regulates the response to S1P. Moreover, sphingosine kinase 1, the enzyme responsible for the phosphorylation of sphingosine forming S1P (Figure 2), is up-regulated, suggesting that OPCs modulate their own production of S1P, at least in part, through GPR17 expression and/or signaling [98].

The recruitment of OPCs following CNS demyelination can also lead to the generation of myelin-forming Schwann cells, both in animal models and in MS patients [79,101,102]. The contribution of this event to myelin regeneration should not be ignored, even if the mechanisms driving OPCs to differentiate into Schwann cells rather than oligodendrocytes remain unclear, as is whether this could represent an interesting opportunity from a therapeutic point of view.

A fraction of adult OPCs seems to be generated from subventricular zone (SVZ)-derived progenitors, apparently able to migrate into the lesion areas and to differentiate into myelin-forming OPCs involved in remyelination [79]. However, the relevance of this contribution is still controversial, even if SVZ progenitors might have indirect effects on myelin regeneration.

In addition to new OPC-derived myelinating cells (either oligodendrocytes or Schwann cells), surviving mature oligodendrocytes can form new myelin in lesion areas. Mature oligodendrocytes were long considered too far along the differentiation process to maintain any plasticity or regeneration potential, although it had been suggested that they might play indirect roles in the remyelination process. However, recent studies have clearly demonstrated that new myelin can be generated by spared mature oligodendrocytes in animal models [103-105], but to a limited extent [106]. Conversely, a study in MS patients showed that newly generated oligodendrocytes were present in remyelinated lesions only in very small numbers, suggesting that the contribution of spared oligodendrocytes to myelin regeneration might be more prominent in humans [107,108].

### Paving the road to myelin regeneration

In demyelinated lesions, the cascade of events leading to the formation of new myelin, mainly by the differentiation of adult OPCs but with possible contributions from other cell types, is sustained, at least initially, by the sensing of tissue damage by the innate immune response. This first event, mainly due to recruitment and activation of microglia, is followed by a complex network of interactions between different cellular populations, both resident in the CNS or recruited to the lesion site from the periphery. At the same time, the response to tissue damage triggered by microglial activation leads to a series of events that are unfavorable for spontaneous myelin renewal, the most evident of which being the powerful chronic inflammatory response present in several demyelinating diseases. This review does not aim to dig into the details of this complexity. However, we would like to emphasize that 1) the same cell population can be activated in a dual (or multiple) mode, which can be either inhibitory or faciliatory for myelin regeneration; 2) there is no single cell population, stimulus, or mediator that drives the evolution of the lesion toward myelin regeneration or remyelination failure with subsequent neurodegeneration. Consequently, any approach aimed at removing the obstacles to effective regeneration of damaged myelin would need to target multiple pathways and cell populations [109]. The aim of an effective remyelinating therapy should thus be the switch from detrimental to beneficial patterns at multiple cellular levels.

In response to a demyelination event, microglia, CNS-resident macrophages, are activated and recruited to the lesion site. Microglial activation is without doubt an important direct and indirect (mediated in turn by astrocyte activation) factor eliciting the neuroinflammatory response, both locally and mediated by the infiltration of reactive T cells and macrophages from the periphery. For some diseases, it likely represents the most critical factor opposing myelin regeneration and driving disease progression. On the other hand, the traditional classification of microglia into M1 (proinflammatory) and M2 (immunomodatory, ‘wound-healing’) phenotypes represents a strong oversimplification, considering the emerging vast diversity of microglia subsets under basal conditions and upon disease-specific activation, reflecting a much greater variety of functional roles for this cell population [110,111]. In fact, microglia with protective/regenerative phenotypes are present in many demyelinating diseases (reviewed in [112-114]). Indeed, the transition between the early-stage pro-inflammatory phenotype and a later-stage regenerative phenotype seems to be a crucial permissive event for remyelination and impaired remyelination is associated with chronic pro-inflammatory microglial activation [115]. Possibly the best understood remyelination-permissive function of microglia is the removal of myelin debris, which strongly inhibits OPC differentiation and new myelin formation. Activated microglia effectively phagocytose myelin debris, thus removing this inhibitory cue [114]. The phagocytic ability of microglia is reduced with aging and in MS patients [116], but it can be effectively enhanced by pharmacological treatments, such as the remyelination-inducing antibody rHIgM22 [13]. As discussed below, the administration of rHIgM22 to rat mixed glia cultures alters the balance between ceramide and S1P, suggesting the importance of sphingolipid signaling in the activation of microglia phagocytosis. Additional *in vitro* studies also indicate that the selective S1P receptor agonist siponimod increases the expression of NR4A anti-inflammatory genes in microglia, without affecting the functions of peripheral blood mononuclear cells and oligodendrocytes [117], further highlighting the importance of S1P signaling in driving microglia toward the regenerative phenotype.

Aside from their phagocytic ability to clear debris, activated microglia with a regenerative phenotype release a wide-ranging array of cytokines, growth factors, and other soluble signaling molecules (including S1P [118]) able to influence several aspects of the progression of oligodendrocyte lineage cells toward formation of new myelin [78,119,120]. Microglia-derived signals support migration, proliferation, and differentiation of OPCs (without affecting OPC apoptosis), eventually allowing remyelination, as elegantly confirmed in a recent study analyzing the effects of microglia depletion [121]. Activated microglia may also stimulate remyelination by driving SVZ progenitors toward oligodendrogliogenesis [122]. Finally, activated microglia contribute to the remodeling of the extracellular matrix, rendering it more permissive to OPC differentiation [123].

Like microglia, astrocytes are highly sensitive to local changes associated with tissue lesions, including demyelination, and, as is the case for microglia, strong and sustained activation of astrocytes is detectable in lesions in almost all demyelinating diseases, including in MS [124]. Moreover, similarly to microglia, at least two different activated astrocyte phenotypes have been described, the neurotoxic phenotype A1 and the neuroprotective phenotype A2, although recent studies also revealed a much higher phenotypic heterogeneity [125]. In MS, astrocyte activation contributes to the recruitment of immune system cells from the periphery 1) by modulating the integrity of the blood–brain barrier and remodeling the extracellular matrix and 2) by producing a plethora of chemokines and cytokines which affect migration of peripheral immune cells to the CNS and/or their commitment to a pro-inflammatory phenotype [126]. The release of soluble mediators also contributes to the activation of CNS-resident immune system cells, microglia, and macrophages. In turn, soluble factors and extracellular vesicles released by activated microglia contribute to pro-inflammatory activation of astrocytes [127].

Astrocyte activation thus contributes to the inflammatory response, but it also plays a crucial role in isolating the damaged area and containing the spread of inflammatory responses and of tissue destruction via the formation of a glial scar, which is consistently observed in MS patients and in MS animal models. The scar isolates the damaged area, preventing the spread of tissue destruction; however, glial scar inflexibility also results in inhibition of remyelination and axon regeneration [128-130]. In addition to their effects on the inflammatory response, activated astrocytes have direct toxic effects on neurons and oligodendrocytes and exert detrimental effects by blocking the proliferation and differentiation of OPCs [126,131].

In contrast, alternative astrocyte activation (very likely toward the neuroprotective A2 phenotype) within lesions can limit the detrimental effects of pro-inflammatory factors, thus providing support and protection for oligodendrocytes and neurons [126,132]. Astrocytes also play important roles in maintaining the homeostasis and spatial distribution of different secreted factors that determine OPC proliferation, migration, and differentiation, thereby directly affecting spontaneous remyelination in demyelinating diseases [126,132]. Of the protective factors (energy substrates and trophic factors) produced by cytokine-activated astrocytes that exert beneficial effects on remyelination, and in general promote recovery of CNS function after lesion, some highlight the importance of S1P signaling for myelin regeneration:

- The chemokine CXCL1 is produced in reactive astrocytes (stimulated with the pro-inflammatory cytokine IL1β) and binds the CXCR2 receptor expressed by oligodendrocytes. CXCL1 produced by hypertrophic astrocytes in MS could represent a mechanism for recruitment of oligodendrocytes to the damaged area, a prerequisite for remyelination [133,134].
- Sphingosine kinase 1 and S1P_3_ are up-regulated on reactive astrocytes in MS lesions and in cultured rat astrocytes treated with the pro-inflammatory molecule LPS. S1P induces secretion of CXCL1 in astrocytes, and this is amplified in astrocytes pretreated with LPS. Thus, activation of the SK1/S1P_3_ pathway in astrocytes could be both detrimental, by enhancing astrogliosis, and beneficial, through increased remyelination sustained by CXCL1 [62].
- IL-11 is up-regulated in astrocytes in MS lesions. IL-11 enhances oligodendrocyte survival and maturation and increases myelin formation in rodent CNS co-cultures [135-138]. Remarkably, production and secretion of IL-11 in astrocytes is induced by the S1P analog fingolimod interacting with S1P_1_ and S1P_3_ receptors [139].

Indeed, S1P signaling in astrocytes (and in microglia) seems to be relevant to its unexpected beneficial effects on myelin regeneration in MS (Reviewed in [63,139]). As mentioned above, fingolimod is effective in MS by blocking the migration of immune cells, with consequent redistribution of T cells to secondary lymphoid organs and prevention of invasion of auto-aggressive T cells to the CNS. However, S1P receptors are widely expressed in the CNS, fingolimod easily crosses the BBB, and the effect on MS is at least in part independent of the effect on the migration of immune cells from the periphery [84]. In fact, emerging evidence indicates that fingolimod has direct effects in the CNS in MS. S1P signaling effects relevant for MS likely involve multiple neural cell types (astrocytes, oligodendrocytes, neurons, microglia, and dendritic cells); however, very strong evidence points out the importance of astrocytes in the direct CNS effects of fingolimod [84,140-143]. Astrocytes express S1P receptors, mainly S1P_1_ and S1P_3_, S1P_1_, and S1P_3_ are up-regulated in reactive astrocytes present in demyelinating and chronic MS lesions [63], and S1P modifies astrocyte morphology and increases expression of GFAP, a marker of astrogliosis. *In vitro*, fingolimod stimulates astrocyte migration, while *in vivo* it acts as a functional antagonist of astrocyte S1P_1_. In EAE, fingolimod is highly effective but its efficacy is lost in animals deficient in S1P_1_ selectively in astrocytes. As an endpoint, fingolimod appears to be able to promote remyelination by acting on oligodendrocytes, microglia or astrocytes [143,144].

In addition to their direct effects on inflammation in demyelinating lesions, astrocytes (activated by microglia) could in turn affect microglia function, by recruiting microglia to the lesion sites and stimulating their phagocytic activity [145]. Remarkably, activated astrocytes themselves also seem to be able to remove myelin debris [146].

Faced with the complexity of interactions between the various cell types, developing effective remyelination-promoting therapies seems a daunting task. However, such therapies are currently under development, and some appear to be able to act in a coordinated manner on multiple cellular targets involved in the process of myelin regeneration. As an example, we have been investigating the use of the recombinant human antibody IgM22 (rHIgM22) [118,147]. rHIgM22 is a recombinant form of a human monoclonal IgM [148] that is a naturally occurring antibody belonging to a family of immunoglobulins produced by B-cells in a T-cell-independent manner in the absence of a specific foreign antigen. The functions of these antibodies are not fully understood, although it is assumed that they play protective roles as part of the innate immune system. rHIgM22 treatment effectively enhances myelin regeneration in both immune and non-immune mouse models of demyelination [149-152]. Only mature oligodendrocytes show significant surface binding of rHIgM22; however, rHIgM22 is able to induce 1) an increase in intracellular calcium in astrocytes, OPCs, and oligodendrocytes at different stages of differentiation, although with distinct kinetics [153]; 2) OPC proliferation in rat mixed glial cells (MGCs), but not in purified OPC cultures [154], suggesting that its effects on OPCs might require the activation of other cell populations present in the mixed culture; 3) myelin debris phagocytosis by microglia [13].

rHIgM22 induces proliferation in MGCs, with the most significant response associated with astrocytes, and increases the production and release of S1P by microglia, suggesting that rHIgM22 indirectly influences astrocyte proliferation via microglia-released S1P. The sphingolipid rheostat, i.e. the fine balance between the signaling sphingolipids ceramide and S1P, seems to be an important target of rHIgM22 action. In fact, rHIgM22 treatment in differentiated oligodendrocytes reduces the activity of ASM, a key mediator for the detrimental effects of ceramide observed in mouse models of MS (genetic or pharmacological inhibition of ASM is protective against lesions in mouse models of MS [54,55,57]). rHIgM22 has no effects on glycosphingolipids in MGCs and pure astrocytes, while in OPCs, oligodendrocytes, and microglia there is a significant increase in the levels of GM3 and GD3 gangliosides (Prinetti A., Grassi S., Prioni S. unpublished results). Thus, we hypothesize that rHIgM22’s myelin repair activity is mediated by alterations of lipid-dependent membrane organization in glial cells, which requires the orchestration of the responses between different sphingolipid mediators in the sphingolipid rheostat in multiple cellular populations in the lesion niche.

### Concluding remarks

Demyelination is a common feature of several diverse diseases. In the case of acquired demyelinating diseases (of which MS is the most prevalent), their etiopathogenesis is complex, multifactorial, and not yet fully understood. Sustained neuroinflammation is the hallmark of many of these diseases. For MS, autoimmunity is regarded as the cause of the disease. Genome-wide association studies corroborate the relevance of the immune component for the onset and progression of the disease. Several environmental risk factors appear to exacerbate the neuroinflammatory condition, and anti-inflammatory therapies are valuable disease-modifying treatments. However, inflammation is clearly only part of the picture, and variations of genes expressed in CNS-resident cells highlight the importance of neuroprotective and regenerative mechanisms in reducing the severity of the disease.

The adult brain can react to myelin damage and disruption by triggering myelin regeneration. However, effective myelin regeneration requires 1) the removal of *inhibitory conditions* at the lesion site; 2) the convergence of several different molecular clues generating *permissive conditions* for myelin regeneration; 3) the effective recruitment of myelin-forming cells. In this review, we outlined the orchestrated contribution of different cell populations to these events.

The activation and recruitment of different cell types contributing to the three conditions mentioned above requires a complex network of signals, and focusing on a specific pathway would be naïve and reductive. However, the contribution of sphingolipid-dependent signaling to the onset and evolution of demyelinating diseases on one side, and to the process of myelin regeneration on the other, is remarkable. Mutations in the *GALC* gene, encoding the glycolipid catabolic enzyme galactocerebrosidase, represent a major genetic risk factor for the onset of MS as they prevent microglia from effectively removing myelin debris, thus hampering myelin regeneration.

Ceramide is detrimental for the onset and progression of MS. Ceramide generation in reactive astrocytes affects oligodendrocyte survival and worsens demyelination [52]. ASM inhibition is a realistic target since it can be achieved with commonly prescribed antidepressant drugs [53-57]. However, enzymes implicated in the ceramide biosynthetic pathway have also received attention recently. Ceramide generation in astrocytes with detrimental effects can be mediated by ceramide synthase 2 [59], while ceramide synthases 5 and 6 in neurons seem to be involved in the progression of EAE [60]. Thus, addressing ceramide metabolism for therapeutical purposes might need to not only target several enzymes but multiple cell populations as well.

Even more complex and intriguing is the role of S1P. S1P drives peripheral lymphocytes to the CNS. This can be prevented by antagonizing S1P_1_ receptors in lymphocytes using fingolimod [61,62], however, fingolimod requires the engagement of S1P_1_ in astrocytes [61,63] and can exert protective effects by inducing the activation of microglia [117]. On the other hand, the development of selective S1P receptor modulators recently highlighted the direct impact of S1P signaling on myelin regeneration, either by favoring the survival of oligodendrocytes [86,87] or by driving the differentiation of OPCs [88].

If targeting specific molecular and cellular aspects involved in myelin regeneration represents a straightforward therapeutic option, remyelination-inducing drugs might be even more effective if they simultaneously address more than one target, as exemplified above in the case of rHIgM22 [155].

**Perspectives**

- About 3 million people worldwide live with a demyelinating condition (this is likely heavily underestimated due to the lack of data from some areas of the world). Thus, treatments aimed at preventing myelin loss and its consequences or at improving the effectiveness of myelin regeneration are a major therapeutic need. The cure for demyelinating diseases would be myelin regeneration. Myelin regeneration seems to be the body’s standard response to myelin loss; however, its efficacy in human patients is impaired for several reasons. Effective myelin regeneration requires the orchestrated intervention of different cell populations aimed at creating a permissive environment for the recruitment of myelin-forming cells.
- The levels of different bioactive sphingolipids (ceramide and S1P) are altered in several demyelinating diseases and altered sphingolipid signaling affects both myelin loss and myelin regeneration. The production of ceramide via sphingomyelin hydrolysis or de novo synthesis is responsible for increased oligodendrocyte death and neurodegeneration. The production of S1P has a dual valence, on the one hand triggering pro-inflammatory responses, and, on the other, driving the release of signals by microglia and astrocytes that positively affect the recruitment of myelin-forming cells. Moreover, S1P signaling directly affects myelin regeneration by exerting protective effects on degenerating oligodendrocytes or by boosting OPC differentiation, depending on the specific S1P receptor engaged.
- Treatments able to affect sphingolipid metabolism and signaling hold promise to effectively improve the brain’s capability for myelin regeneration. The dissection of the contribution of specific sphingolipid-mediated events in different cell populations involved in myelin regeneration is needed to develop therapies able to address the complexity of human demyelinating diseases.

## Open Access

This article has been published open access under our Subscribe to Open programme, made possible through the support of our subscribing institutions, learn more here: https://portlandpress.com/pages/open_access_options_and_prices#conditional

## Abbreviations

AD: Alzheimer’s disease
ASM: acid sphingomyelinase
CNS: central nervous system
EAE: experimental autoimmune encephalomyelitis
GALC: galactocerebrosidase
MGC: mixed glial cells
MS: multiple sclerosis
OPC: oligodendrocyte precursor cell
RR-MS: relapsing-remitting multiple sclerosis
S1P: sphingosine 1-phosphate
rHIgM22: recombinant human IgM22
